# A Novel Bioengineered Functional Motor Unit Platform to Study Neuromuscular Interaction

**DOI:** 10.3390/jcm9103238

**Published:** 2020-10-10

**Authors:** Jasdeep Saini, Alessandro Faroni, Adam J. Reid, Kamel Mamchaoui, Vincent Mouly, Gillian Butler-Browne, Adam P. Lightfoot, Jamie S. McPhee, Hans Degens, Nasser Al-Shanti

**Affiliations:** 1 Musculoskeletal Science & Sports Medicine Research Centre, Department of Life Sciences, Manchester Metropolitan University, Manchester M1 5GD, UK; JASDEEP.SAINI@stu.mmu.ac.uk (J.S.); a.lightfoot@mmu.ac.uk (A.P.L.); h.degens@mmu.ac.uk (H.D.); 2 Manchester Academic Health Science Centre, Blond McIndoe Laboratories, Division of Cell Matrix Biology and Regenerative Medicine, Faculty of Biology Medicine and Health, School of Biological Sciences, University of Manchester, Manchester M1 7DN, UK; alessandro.faroni@manchester.ac.uk (A.F.); Adam.Reid@manchester.ac.uk (A.J.R.); 3 Manchester Academic Health Science Centre, Department of Plastic Surgery & Burns, Manchester University NHS Foundation Trust, Wythenshawe Hospital, Manchester M23 9LT, UK; 4 Center for Research in Myology, Sorbonne Université-INSERM, 75013 Paris, France; kamel.mamchaoui@upmc.fr (K.M.); vincent.mouly@upmc.fr (V.M.); gillian.butler-browne@upmc.fr (G.B.-B.); 5 Department of Sport and Exercise Sciences, Manchester Metropolitan University, Manchester M1 5GD, UK; J.S.McPhee@mmu.ac.uk; 6 Institute of Sport Science and Innovations, Lithuanian Sports University, LT-44221 Kaunas, Lithuania

**Keywords:** motor unit (MU), neuromuscular junction (NMJ), motor neuron (MN) co-culture, myotube, human myoblast

## Abstract

Background: In many neurodegenerative and muscular disorders, and loss of innervation in sarcopenia, improper reinnervation of muscle and dysfunction of the motor unit (MU) are key pathogenic features. *In vivo* studies of MUs are constrained due to difficulties isolating and extracting functional MUs, so there is a need for a simplified and reproducible system of engineered *in vitro* MUs. Objective: to develop and characterise a functional MU model *in vitro*, permitting the analysis of MU development and function. Methods: an immortalised human myoblast cell line was co-cultured with rat embryo spinal cord explants in a serum-free/growth fact media. MUs developed and the morphology of their components (neuromuscular junction (NMJ), myotubes and motor neurons) were characterised using immunocytochemistry, phase contrast and confocal microscopy. The function of the MU was evaluated through live observations and videography of spontaneous myotube contractions after challenge with cholinergic antagonists and glutamatergic agonists. Results: blocking acetylcholine receptors with α-bungarotoxin resulted in complete, cessation of myotube contractions, which was reversible with tubocurarine. Furthermore, myotube activity was significantly higher with the application of L-glutamic acid. All these observations indicate the formed MU are functional. Conclusion: a functional nerve-muscle co-culture model was established that has potential for drug screening and pathophysiological studies of neuromuscular interactions.

## 1. Introduction

Neuromuscular junctions (NMJs) consist of presynaptic motoneuron terminals and postsynaptic motor endplates located on skeletal muscle fibres. The NMJ is required for the transmission of electrical signals from the nervous system to be ultimately transformed into skeletal muscle contraction [1]. At the NMJ, an action potential from a motoneuron reaches the presynaptic terminal, inducing release of acetylcholine (ACh) into the synaptic cleft. The binding of ACh to postsynaptic Ach receptors on the skeletal muscle motor endplate initiates depolarisation along the sarcolemma that induces calcium release from the sarcoplasmic reticulum to enable cross-bridge cycling and hence skeletal muscle contraction [2]. Loss of motor neurons and/or defects in the NMJ result in paralysis of the muscle [3]. Consequently, neuromuscular diseases (NMD) such as myasthenia gravis [4] are accompanied of severe skeletal muscle weakness and wasting and/or early onset of muscle fatigue. Additionally, the diseases and age-linked loss of muscle mass and function are associated with denervation through the loss of motor neurons [5] and postsynaptic disintegration of acetylcholine receptors (AChRs). Despite the fact that degeneration of the NMJ is a fundamental aspect of neurodegenerative diseases and muscle wasting associated with ageing and diseases, existing methods to investigate the specific contribution of degeneration of the NMJ to the aetiology of such conditions are limited [6]. Given the importance of innervation and functional NMJs for muscle differentiation and function [7], models and methods that enable the analysis and modulation of NMJs may considerably enhance understanding of NMD pathogenesis and provide a platform for the validation of new treatments. To address the issue of NMJ formation through functional innervation, several murine and rodent studies have been reported [8]. However, they are fundamentally a poor surrogate for human biology, in particular in terms of MU (motor unit) or NMJ mechanistic studies and drug discovery with no real translational impact being felt in the last decade or so. Moreover, the study of the MU from a mechanistic perspective is challenging in *in vivo* models. To elucidate potential mechanisms and pathways, there is a need to develop a robust and physiologically relevant model that contains all the components of MU to overcome the limitation of existing animal models in studying NMJ development and physiological function. Few contemporary nerve-muscle co-culture systems have been produced using human embryonic stem cells induced pluripotent stem cell and cross species systems using co-culture of primary human myoblasts with mouse or rat neuronal cells [9].

Furthermore, the reduction and replacement of animal use in research should be a target for all researchers. An alternative is cell culture, but most *in vitro* models to study the neuromuscular system are monocultures of skeletal muscle cells (SkMCs), frequently of animal origin [10,11], and the absence of functional innervation and lack of NMJ formation in these models result in an incomplete replication of *in vivo* conditions [12]. Given the importance of innervation and functional NMJs for muscle differentiation and function [13], models where NMJs are generated may considerably enhance understanding of neuromuscular pathologies and sarcopenia and provide a platform for the validation of new treatments.

To address the issue of NMJ formation through functional innervation, nerve-muscle co-culture systems have been produced using human embryonic stem cells [14] and induced pluripotent stem cells [15]. In addition, cross-species systems co-culturing primary human myoblasts with mouse or rat neuronal cells have been developed [16,17,18]. However, the intricate nature of these systems results in large variations in experimental procedures and outcomes. For instance, the inclusion of sera (e.g., fetal calf) introduces indeterminate variables to the system due to differences in serum composition between samples [19], which may influence the results of experimental interventions and hence hamper reproducibility. In fact, there is some evidence that serum employed in these systems produces retarded motor neuron myelination *in vitro* [20]. Furthermore, the use of primary myoblasts acquired from muscle biopsies has its own distinctive limitations in co-culture systems. Primary myoblasts have a limited capacity for expansion and undergo phenotypic alterations, including senescence through successive cell expansion, as well as becoming a less homogeneous cell population [21,22]. Recent advances in the application of cells derived from embryonic stem cells and induced pluripotent stem cells to generate myoblasts [23], and motor neurons may overcome some of these limitations [24]. However, besides ethical issues of using human embryonic stem cells, motor neurons derived from stem cells are difficult to culture and require media formulations with neurotrophic factors that interfere with SkMC differentiation [25]. Furthermore, co-cultures of myoblasts with stem cell-derived motor neurons produce unstable NMJs that are not suitable for longer studies [25]. Moreover, these models use complicated cocktails culture media that contain more than 15 neural growth factors. This further complicates drug discovery and toxicology studies due to possible cross-communication of the novel compound with factors contained within the added media, possibly explaining why many promising therapies do not translate to clinics [26]. Recently, we have developed a co-culture of rat embryonic spinal cord explants with primary human myoblasts resulted in NMJ formation and enhanced differentiation of myotubes that was stable for at least 2 weeks without the need of serum or neural growth factors [27].

The aim of the present study was to establish, characterise and functionally assess an easily reproducible MU co-culture system. The co-culture system was established using streamlined methods and did not require serum and growth/neurotrophic factors, improving experimental reproducibility. Motor neurons were generated using spinal cord explants sliced from rat embryo spinal cord. The explants were used to innervate immortalised human myoblasts, which were simultaneously being differentiated to myotubes. Here, we show that this co-culture system resulted in NMJ formation with the co-localisation of motor neuron axon terminals with AChR accumulations on differentiated contractile myotubes. The formation of functionally active NMJs presenting with mature pre/post synaptic characteristics with the typical twisting perforated structure was also observed. This simplified co-culture system thus presents a promising tool to study the mechanisms underlying NMJ dysfunction associated with muscle wasting in ageing and neuromuscular disorders and a platform to trial innovative therapies.

## 2. Experimental Section

### 2.1. Immortalised Human Skeletal Muscle Cell Culture

An immortalised human SkMC line was generated at the institute of Myology (Paris, France). The cell line was established using primary human myoblasts obtained anonymously from Myobank, a tissue bank affiliated to Eurobiobank, which has the agreement from the French Ministry of Research (authorisation # AC-2013-1868). The primary myoblasts originated from biopsies of the semitendinosus muscle of a 25-year-old man, not diagnosed with any genetic defects or disease. Myoblast immortalisation was achieved using transduction with both telomerase-expressing and cyclin-dependent kinase 4-expressing vectors [28]. A cryopreserved suspension of 1 × 10^6^ immortalised human myoblasts was thawed and resuspended in 10 mL of complete growth media (GM) (Table 1), then pipetted into a T75 flask and incubated for SkMC proliferation. The cells were incubated at 37 °C with a 5% CO_2_ atmosphere until the flask was 80% confluent. Subsequently, cells were washed twice with Dulbecco’s Phosphate Buffered Saline (DPBS) from Lonza (Basel, Switzerland). The cells were then disassociated for 5 min in 2 mL of TrypLE™ Express Enzyme from Thermo Fisher Scientific (Waltham, MA, USA) at 37 °C in 5% CO_2_. The 2 mL cell suspension was then transferred into a conical tube and homogenised in 8 mL of GM. The cells were counted and seeded on a 35 mm glass-bottom µ-Dish from Ibidi^®^ (Martinsried, Germany) at a density of 350 cells/mm^2^. After 24 h of incubation, the myoblast density reached 90–100% confluence. The cells were then washed twice with DPBS and incubated for 24 h at 37 °C with a 5% CO_2_ atmosphere in a simplified differentiation medium (DM), consisting of 99% (*v*/*v*) DMEM (Dulbecco’s Modified Eagle Media), 1% (*v*/*v*) L-glutamine, 10 µg/mL recombinant human insulin and 10 µg/mL gentamicin, before plating the rat embryo spinal cord explants.

### 2.2. Isolation of Rat Embryonic Spinal Cord Explants

All animal work undertaken was approved by the Home Office and carried out at the University of Manchester, in accordance with the Animal Scientific Procedures Act 1986 [27]. Time-mated Sprague Dawley rats obtained from Charles River Laboratories (Oxford, UK) were sacrificed with CO_2_ when embryos were roughly embryonic development day (ED) 13.5. The uterine horn was removed from the pregnant rat and transferred to a sterile sample-collection pot containing Hank’s Balanced Salt Solution from Lonza with 10% FBS. Embryo dissection was performed in a 100 mm dish under a binocular microscope using 21-gauge needles. The spinal cord was dissected in one piece from each embryo and the surrounding connective tissue was removed, ensuring the dorsal root ganglia (DRGs) remained intact and attached to the spinal cord. Following removal of connective tissue, the spinal cord was sliced transversally into ~1–2 mm^3^ explants.

### 2.3. Co-Culture

Following 24 h of incubation with DM, the SkMCs were primed for innervation as they were in the initial phases of differentiation, transitioning from myoblast to myocyte, before substantial cell fusion and myotube formation occurred. The DM was removed from the 6-well plate dishes, and the cells were washed twice with DPBS. Then, DM was added to each dish, thinly coating the cells on the glass bottom. Between three and six evenly spaced explants were placed into each dish and incubated for 6 h (under the previously mentioned atmospheric conditions), to allow the explants to adhere with the SkMCs. Following incubation, the explants become slightly affixed to the SkMCs, at which time an additional DM was added dropwise to each dish to prevent dehydration of the SkMCs and spinal cord explants. The cells were then incubated for an additional 24 h before adding a further DM to each dish. While the myocytes fuse into immature myotubes between 24 and 48 h, sprouting neurites from the explants innervate the cells at this stage of development. Co-cultures were maintained by changing half the DM every 48 h. Live cells were visualised using a Leica DMI6000 B inverted microscope from Leica Microsystems (Wetzlar, Germany). Myotube contractions were video captured for 30 s at 24 frames per second with phase contrast microscopy.

### 2.4. Immunocytochemistry

The cells were washed twice with DPBS and fixed in 4% paraformaldehyde for 10 min at 21 °C. The fixed cells were washed thrice with DPBS and permeabilised by incubation with 1x perm/wash buffer from Becton, Dickinson (BD) Biosciences (Franklin Lakes, NJ, USA) for 30 min at 21 °C, followed by washing twice with DPBS. Subsequently, the cells were incubated for 1 h in a blocking solution of 0.2% Triton X-100 with 10% normal goat (GS) or normal donkey serum (DS) all from Sigma-Aldrich. The blocking solution was removed, and the cells washed once with DPBS. The primary antibody diluent consisted of 3% GS or DS with 0.05% Tween-20 from Sigma-Aldrich. The primary antibodies (Table 2) were added to the cells and incubated for 18–24 h at 4 °C. Following primary antibody incubation, the cells were washed thrice with DPBS before incubation with the corresponding secondary antibodies, along with 4′,6-Diamidine-2′-phenylindole dihydrochloride (DAPI) from Sigma-Aldrich for 30 min at 21 °C. Confirmation of myotube innervation and NMJ formation was assessed via confocal and immunofluorescence microscopy using a Leica DMI6000 B inverted microscope and a Leica TCS SP5 confocal microscope from Leica Microsystems.

### 2.5. Assessment of Functional NMJ Formation

Co-cultures were functionally evaluated to validate the formation NMJs via live video analysis of myotube contraction frequency in response to agonist/antagonist treatments. The co-cultured cell dishes were positioned onto a DMI6000 B inverted microscope stage enclosed by an incubation chamber to maintain atmospheric conditions of 37 °C in 5% CO_2_, allowing for spontaneously induced myotube contractions. Following 5 min of sustained spontaneous myotube activity, the cultures were treated with the cholinergic antagonists’ alpha-bungarotoxin (α-BTX) at a dilution of 1:400 or (+)-tubocurarine chloride pentahydrate (DTC, Sigma cat # 93750) at a concentration of 8 µM from Sigma-Aldrich, to block AChRs at the NMJ. Co-cultures were also treated with the glutamatergic agonist L-glutamic acid from Sigma-Aldrich (cat # G1251) at a final concentration of 400 µM to stimulate glutamate receptors on the motor neurons. The specified concentrations where selected based on previously established studies [29,30,31]. Myotube contraction frequency was measured 30 s before treatment to determine spontaneous baseline contractile activity. Subsequently, contraction frequency was measured immediately upon addition of the treatment to the cells and then measured again after 1 min, 2 min, 5 min, 10 min, 30 min and 1 h. After 1 h, the cells were washed twice with DPBS and fresh untreated DM was added to the cells. Contraction frequency was measured again immediately following washout and resupply of DM, then again at 1 h 30 min and 24 h after the initial treatments. Live video analysis was conducted at 24 frames per second with phase contrast microscopy. Myotube contraction frequency was expressed as a mean ± standard deviation using the equation that Hertz = 60 cycles per minute.

### 2.6. Statistical Analyses

The results presented from the experimental outcomes of this work are representative of a minimum of three independent experiments. Results were analysed using GraphPad Prism v6.05 statistical analysis software. Data was expressed as mean plus/minus standard deviation (± SD). Statistical differences were analysed with unpaired t test. Statistical significance was accepted if *p* < 0.05.

## 3. Results

### 3.1. Co-Culture Morphological Characterisation

**Figure 1:**
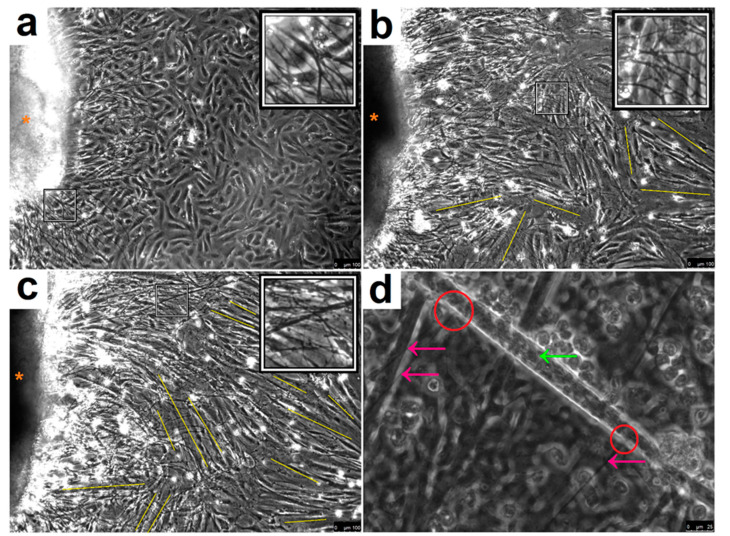
Immortalised human skeletal muscle cells co-cultured with rat embryo spinal cord explants. (**a**) Phase contrast image of explant (orange star) sprouting neurites (shown in enlarged inset) after 24 h over undifferentiated myocytes. Image captured at 10x magnification. Scale bar: 100 µm. (**b**) Multinucleated myotube formation (indicated with yellow lines) after 48 h with continued expansion of neural projections (shown in enlarged inset) emanating from the spinal cord explant (orange star). Image captured at 10x magnification. Scale bar: 100 µm. (**c**) Maintained neurite growth (shown in inset) and continued myotube formation (yellow lines) at 72 h. Image captured at 10x magnification. Scale bar: 100 µm. (**d**) Neuronal axons (pink arrows) form a visible link (circled in red) with a myotube (green arrow). Image captured at 40x magnification. Scale bar: 25 µm.

**Figure 2:**
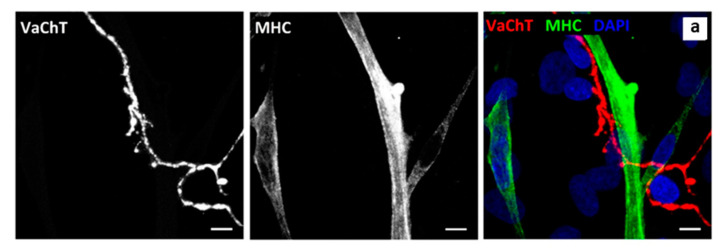
Cholinergic motor neurons co-localise with myotubes at Day 14. Panel a is a representative image of co-culture stained for vesicular acetylcholine transporter (VaChT) (red), myosin heavy chain (MHC) (green) and DAPI (blue). Scale bar = 7.5 µm.

Myoblasts were co-cultured in a simplified DM with spinal cord explants from embryonic development day (ED) 13–14 rat embryos (Figure 1). At 24 h, SkMC fusion was absent and the cells displayed typical characteristics of mononucleated myocytes, indicating the cells were still in the initial stage of differentiation (Figure 1a). Successfully adhered explants sprouted neurites and expanded over the SkMCs. At 48 h, clear SkMC differentiation had commenced, and neurite length expanded further to 962 µm ± 57, (Figure 1b). After 72 h, neurite growth expanded to 1503 µm ± 148, with progressive myotube maturation (Figure 1c). Importantly, connections of motor neuron axon terminals with myotubes were visible (Figure 1d and Figure 2) and the first spontaneous contractions of individual myotubes were observed.

### 3.2. Spontaneous Myotube Contractions

The first spontaneously contracting myotubes were observed ~72 h after co-culture, while no contractions were observed in aneurally cultured myotubes which confirm our previous observation [27], as myotubes matured in the co-culture, the number of contracting myotubes and their contraction frequency increased. After seven days in co-culture, the myotubes were contracting continuously in a systematic pattern as a unified network (see Video S1), behaving as an individual motor unit prompted by motor neuron stimulation.

### 3.3. Characterisation of Neuronal Cells

**Figure 3:**
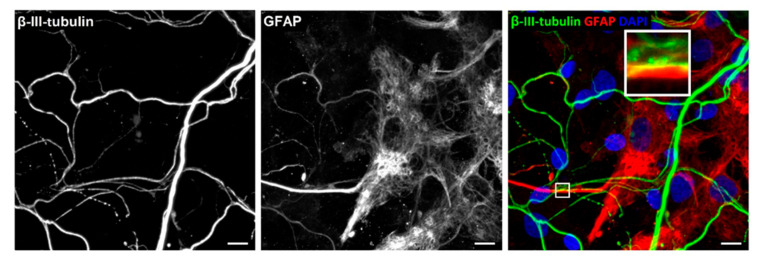
Interaction between neuronal axons and non-myelinating Schwann cells at Day 14. Image is representative of neuronal cells in the co-culture stained for β-III-Tubulin (green), Schwann cells stained for glial fibrillary acidic protein (GFAP) (red) and DAPI (blue). Enlarged Inset shows cellular interaction. Scale bar = 10 µm.

**Figure 4:**
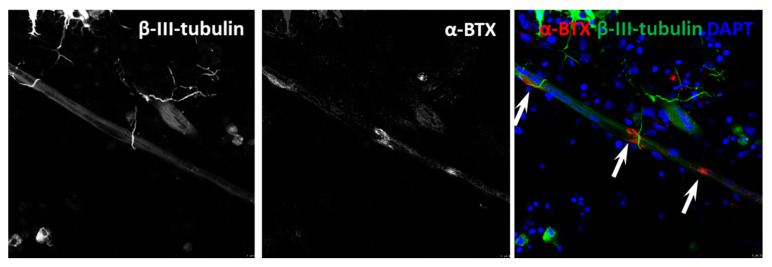
Characterisation of neuromuscular junction formation at Day 14. Representative image of co-culture stained for β-III-tubulin (green), alpha-bungarotoxin (α-BTX) (red) and DAPI (blue). Scale bar = 5 µm. White arrows indicate the NMJ formation and the multiple innervation.

NMJ formation begins with the convergence of cholinergic motor neurons and SkMCs [32]. Therefore, the primary intention was to confirm cholinergic motor neurons and skeletal muscle myotube co-localisation in culture. Confirmation of co-localisation was achieved via antibody staining. Both choline acetyltransferase (ChAT), a cytoplasmic transferase enzyme found in elevated concentration in cholinergic neurons [33], and vesicular acetylcholine transporter (VaChT), a functional mediator of ACh storage and transport by synaptic vesicles [34], were stained to reveal cholinergic motor neurons. Staining myotubes for myosin heavy chain (MHC) was used as an indicator of SkMC differentiation (Figure 2). Staining revealed an abundance of cholinergic motor neurons, with axons terminating at differentiated myotubes. Axon terminals were also seen forming several contact points with individual myotubes, comparable to observations of embryonic innervation. *In vivo*, preliminary myotube innervation occurs via numerous branching axons, which originate from different motor neurons. Maturation causes axon pruning to occur, leaving individual motor neurons to innervate hundreds of mature muscle fibres, generating a functional motor unit [35]. Notably, SkMCs in this co-culture model also exhibit multiple innervation (e.g., 2 or more NMJs per myotube; Figure 3 and Figure 4) similar to what is seen *in vivo* before axon pruning occurs.

Existing co-culture systems focus predominantly on describing the muscle and nerve cells with marginal consideration of associated cells involved with NMJ generation, regulation and development. As mentioned earlier, Schwann cells cap motor nerve terminals *in vivo*. These cells are an essential component in neuromuscular synaptic maintenance and repair [36]. Therefore, revealing Schwann cells in the co-culture system and the subsequent detection of interactions between motor neurons and Schwann cells may explain the robustness of NMJs generated in this co-culture system. Glial fibrillary acidic protein (GFAP), an intermediate filament cytoskeletal component, was used as a marker to reveal the existence of non-myelinating Schwann cells [37]. Motor neurons were distinguished by β-III-tubulin staining, a microtubule cytoskeletal component found in neurons [38] (Figure 3). Co-localisation of neurons and Schwann cells was evident throughout the co-culture. The enlarged inset image in Figure 3 illustrates the interaction between these cells that is indicative of Schwann cells capping axons, as observed *in vivo*. The discovery of co-localisation and cellular interaction between myotubes, motor neurons and Schwann cells within the co-culture supports the concept that this co-culture model provides functional and robust innervation of myotubes via precisely coordinated NMJ formation, mirroring *in vivo* conditions.

### 3.4. NMJ Formation

After validation of co-localisation between cholinergic axon terminals and myotubes, we sought to verify NMJ formation. The formation of NMJs is characterised by the substantial aggregation of AChRs at the myotube membrane in apposition of motor neuron axon terminals. Axon terminals were identified by marking motor neurons for β-III-tubulin (Figure 4) [39]. The accumulation of AChRs on the myotubes was characterised with fluorescently labelled α-BTX, known to bind specifically with AChRs on the myotube membrane [40]. Staining of the co-cultures revealed abundant axon terminals overlying AChRs on myotubes. Comparable to what is seen *in vivo*, AChR clusters exhibited greater concentrations where axon terminals overlapped the clusters, signifying functional NMJ formation. Additionally, diffused fragments of AChRs were speckled on myotube membranes in the absence of axon terminals, indicating that AChRs aggregate in the presence of a nerve terminal.

**Figure 5:**
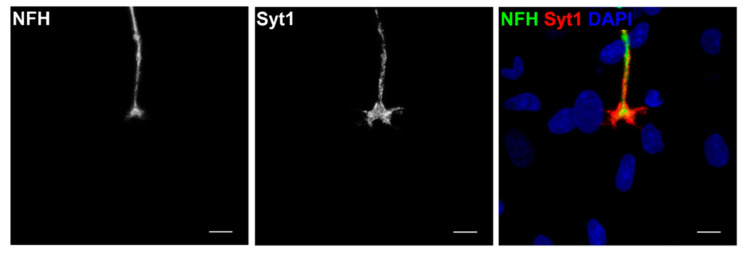
Characterisation of presynaptic neuromuscular junction activity at Day 14. Representative image of co-culture stained for neurofilament heavy (NFH) (green), synaptotagmin (Syt1) (red) and DAPI (blue). Scale bar = 7.5 µm.

Further characterisation of NMJ formation and subsequent functionality was assessed through the examination of the presynaptic apparatus. When an action potential arrives at a presynaptic terminal, the local increase in the Ca2+ concentration triggers the release of ACh. This calcium-dependent exocytosis involves the precise docking of synaptic vesicles to the presynaptic membrane, which is regulated in part by the calcium sensor synaptotagmin (Syt1) [41]. To confirm presynaptic NMJ activity, staining for NFH was used to visualise axons while activity at the terminal was shown by marking Syt1 (Figure 5).

**Figure 6:**
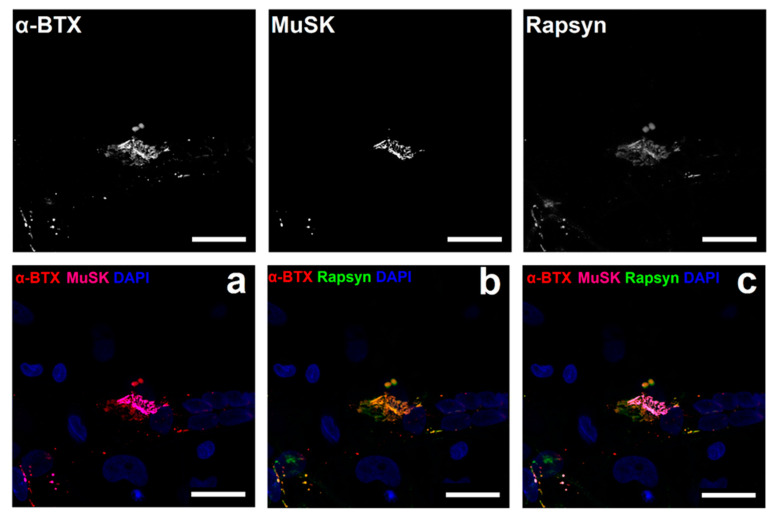
Characterisation of postsynaptic neuromuscular junction formation at Day 14. (**a**) shows co-culture stained for alpha-bungarotoxin (α-BTX) (red), MuSK (magenta) and DAPI (blue). (**b**) is representative of co-culture stained for α-BTX (red), Rapsyn (green) and DAPI (blue). (**c**) reveals interaction and detailed conformation of postsynaptic proteins MuSK (magenta) and Rapsyn (green) at the AChR stained with α-BTX (red) and DAPI (blue). Scale bar = 25 µm.

Subsequent experiments were conducted to identify postsynaptic proteins known to co-localise with AChRs at the endplate. Agrin is crucial for AChR clustering in the postsynaptic membrane and is vital for precise NMJ formation and functions through activation of its muscle-specific receptor tyrosine kinase (MuSK), which forms an initial scaffold for the receptor-associated protein of the synapse (Rapsyn) to advance recruitment of other postsynaptic membrane elements [42]. It was revealed that both Rapsyn and MuSK (Figure 6) precisely overlaid the pretzel shaped structure of the AChR clusters on the postsynaptic membrane.

### 3.5. Functional Assessment

Experiments were conducted to validate the formation of functional NMJs and demonstrate myotube contractions were indeed generated through NMJ formation. Muscle contractions *in vivo* are induced through the presynaptic release of ACh at the NMJ and postsynaptic binding with AChRs. It is well established that α-BTX and DTC inhibit muscle contraction by blocking AChRs at the NMJ *in vivo* [43,44]. Thus, the application of these toxins to this *in vitro* co-culture system should inhibit myotube contractions if functional NMJ formation representative of *in vivo* conditions had occurred. Additionally, it is known that motor neurons *in vivo* are stimulated by glutamatergic neurotransmitters via nervous input through glutamate receptors. The application of glutamatergic agonists *in vitro* increases the generation of action potentials in motor neurons [45,46], leading to an increase of ACh released by the excited motor neurons. Therefore, the addition of the glutamatergic agonist L-glutamic acid (L-Glut) to the co-cultured cells should induce motor neuron excitation resulting in elevated concentrations of ACh at the NMJ and increased myotube contraction frequency.

**Figure 7:**
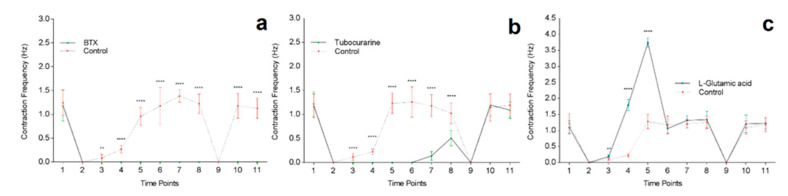
Functional assessment of NMJ formation via the effects of α-bungarotoxin, tubocurarine and L-glutamic acid on myotube contraction frequency at day 14. (**a**) shows the effect of α-bungarotoxin on myotube contraction frequency compared to controls. (**b**) demonstrates tubocurarine effects on myotube contraction frequency compared to controls. (**c**) displays the impact of L-glutamic acid on myotube contraction frequency compared to controls. Data are means ± SD, *n* = 12, each time point analysed with unpaired T-test, ** *p* < 0.01, **** *p* < 0.0001. Time points: (1) -30 s, (2) 0 s, (3) 1 min, (4) 2 min, (5) 5 min, (6) 10 min, (7) 30 min, (8) 1 h, (9) 1 h 1 min (washout), (10) 1 h 30 min, (11) 24 h.

In Figure 7a, the first time point (30 sec before the treatment) shows there is no significant difference in baseline contraction frequency (1.25 Hz ± 0.27 vs. 1.18 Hz ± 0.33, *p* = 0.6) 30 s before treating cells. At the second time point, (0 sec) α-BTX was applied to the cells with equal volume of DM added to the controls, resulting in complete stoppage of all contractions in both control and treated cells, due to changes in environmental conditions from added volume of solution. After one minute of the treatment, myotube contractions in the controls were observable and continued to increase in frequency over time until contraction frequency returned to initial baseline levels after 10 min. However, the α-BTX treated cells exhibited loss of myotube contractility even after washout and media change, indicative of permanent inhibition of AChR function at the NMJ. The cells treated with DTC displayed similar cessation of myotube contractions (Figure 7b). However, 30 min following treatment, contractions were observed in the DTC treated myotubes at a significantly reduced frequency (1.18 Hz ± 0.23 vs. 0.14 Hz ± 0.09, *p* < 0.0001). Contraction frequency increased one hour following treatment, though at a significantly decreased rate compared to controls (1.03 Hz ± 0.22 vs. 0.51 Hz ± 0.16, *p* < 0.0001). Following washout, the DRC treated cells returned to baseline contraction frequency (1.15 Hz ± 0.28 vs. 1.20 Hz ± 0.23, *p* = 0.7) 1 h and 30 min after initial treatment was applied to the cells. Contrasting an irreversible receptor antagonist such as α-BTX, DTC does not inactivate the receptor but decreases the potential of ACh activating it. Spontaneous contraction activity was restored following DTC dissociation from binding sites and clearance from AChRs. These findings demonstrate that myotube contractions were driven by ACh binding with postsynaptic AChRs at the NMJ, which can be abated by blocking the AChRs in this co-culture system.

In contrast to AChR blockers, that halt contractions by acting directly on the postsynaptic myotube motor end plate, L-glut was used to stimulate motor neurons and augment the presynaptic release of ACh, leading to increased contraction frequency (Figure 7c). There was no significant difference between baseline contraction frequencies before the application of L-Glut to the co-cultures. However, comparable to BTX and DTC treatments, there was an immediate cessation of myotube contractions when the treatment diluent or DM was added to the cells (Figure 7). One minute after the application of L-Glut, myotube contraction frequency was slightly yet significantly higher than controls. After two minutes, the treated cells were contracting at a significantly increased frequency of 1.5 Hz higher than the controls. When contraction frequency was measured at 5 min the treated cells increased frequency further to over 2.5 Hz higher than controls. However, after 10 min, the contraction frequency in the treated cells retuned to baseline spontaneous activity similar to controls, where it remained for the duration of the experimental timeline. These findings show that myotube contractions in this co-culture system are instructed through functional NMJ formation.

## 4. Discussion

In order to provide new avenues that facilitate the study of NMJ in development, disease progression to therapeutic intervention, appropriate NMJ experimental models are therefore needed. In the present study, we extended our recent work [27] to fully characterise the NMJ in a rat spinal cord segment and primary human muscle cells co-culture and assess its contractile functionality. The advantages of this platform are (1) the presence of supporting Schwann and glial cells may possibly enhance NMJ robustness and improve function of motor neurons *in vitro* and (2) the formation of functional NMJs that are associated with highly differentiated and motor neuron-driven contracting myotubes.

The co-culture model has advantages over previous skeletal muscle cell cultures as a research tool for investigating neuromuscular and muscle wasting disorders. Firstly, aneurally cultured SkMCs did not spontaneously contract in culture nor did they express the morphological characteristics of innervated SkMCs. However, as similarly occurs *in vivo*, innervated myotubes exhibited endogenously stimulated contractions [47]. Previous research has shown that treating aneurally cultured human myotubes with secretome from rat-nerve/human-muscle co-cultures resulted in a negligible increase of AChR clustering on the myotube [48,49] with no spontaneous contractile activity. This finding suggests that neuronal cell secretions alone are unable to induce *in vitro* contractile function in myotubes, emphasising the requirement of NMJ formation for appropriate development of mature myotubes, which is thus, more representative of the *in vivo* environment.

Furthermore, it has also been demonstrated that denervated myotubes within a nerve-muscle co-culture system fail to exhibit contractile function or characteristics of mature development; they eventually deteriorate, despite the elevated concentration of nerve-derived secretions in the culture environment [50]. Interestingly, the myotubes in our co-culture model initiated spontaneous contractions as early as 72 h post co-culture [27], suggesting the initial emergence of NMJ formation, due to the myotube requirement for nervous input from the motor neurons to induce contraction [51]. When compared to aneural *in vitro* SkMC cultures, the benefits of our co-culture system are apparent, as co-cultured myotubes exhibit advanced stages of differentiation that monocultures of SkMCs fail to achieve. Thus, making this co-culture system suitable for the accurate elucidation of skeletal muscle wasting conditions and development of therapeutic approaches that can mitigate, prevent or ultimately counteract skeletal muscle wasting and weakness of a neural origin. The co-culture model was established using a modified culture media devoid of serum, neurotrophic factors and growth factors. We are not aware of any other models that have generated functional NMJs without supplementation or media enrichment with growth factors [15,30,52,53]. Our simplified approach is easily repeated and diminishes experimental variability thereby improving research potential for drug discovery and to investigate mechanisms responsible for innervated differentiation of myotubes and mature formation of NMJs.

Methods applied throughout this study were designed to optimise the time required for spontaneous myotube contractions while producing functional NMJs [27]. Previously established nerve-muscle co-culture models involve intricate methods requiring various culture media formulations for separate myotube or motor neuron differentiation for at least 10 days before co-culturing [30]. While other studies have presented NMJ formation at 21 days [15]. These lengthy protocols lead to avoidable postponements and possible unintended variation to experimental procedures. In our model, individual myotubes begin to contract by Day 3. The contraction frequency increased, and synchronous unified contractions were apparent by Day 7. To ensure the occurrence of appropriate and mature NMJ formation, the co-cultures were maintained by changing half the culture media on alternating days, with characterization conducted on Day 14. Although co-cultures were characterized on Day 14, preliminary viability experiments were conducted and revealed long-term co-culture studies were possible with this co-culture system, as myotube contractions were observed until experimental termination on Day 30. Notably, the innervated myotubes had the potential for further viability had we continued to maintain the culture beyond 30 days. Some established nerve-muscle models have been considered unsuitable for long-term investigation as they only expressed preliminary characteristics of NMJ formation and lack extended viability [29], though more recent culture systems have shown sustained viability, yet only generating immature NMJs [52].

Myoblasts were cultured with explants consisting of bare ventral horn without DRGs and spinal cord mechanically dissociated to create a neuronal cell suspension for culture with myoblasts. Both these conditions resulted in delayed initiation of spontaneous myotube contractions, increased arrhythmic contractions, reduced contraction frequency and diminished NMJ formation with a poorly developed or failed junction assembly. This suggests motor and sensory neurons originate from both the ventral horn and dorsal root function collectively to innervate myotubes and form NMJs, representative of an *in vivo* environment [54]. Additionally, explants with intact DRGs contain a range of progenitor and supporting cells types. Vital functions such as NMJ formation and the ability to re-innervate the muscle are enhanced by Schwann cells [55]. Therefore, the existence of Schwann cells may encourage NMJ functionality and improved motor neurons function *in vitro*.

The heterogeneous composition (Schwann cells, motor neurons and muscle fibers) of this co-culture model also enhances its applicability as a tool for the precise identification of species-specific pre- or postsynaptic proteins, as the neuronal components in the model are generated from rat and the muscle components from human. Research conducted using a rat-human nerve–muscle co-culture model was developed to investigate the origins of acetylcholinesterase (AChE) in functional NMJs. By exploiting species-specific immunocytochemical staining against AChE, the investigators were able to determine the source of AChE from either the rat nerve cells or the human SkMCs at the synaptic cleft [56]. Importantly, our rat-human co-culture model is devoid of any potential contaminating sera and growth—presenting a novel and native, ex vivo/*in vitro* platform.

The most important observation of this study was that the treatment of the model with several pharmacological agents showed that the spontaneous myotube contractile activity was indeed driven by MN signalling through the NMJ. Functionality of NMJs was assessed through the impact of agonist and antagonist pharmacological interventions that act presynaptically or postsynaptically. The introduction of α-BTX to the co-cultures resulted in an immediate and permanent cessation of the myotube contractions, indicating that the spontaneous contractions were elicited via activation of the AChRs at the NMJ motor endplate, similar to that seen in *in vivo* mammalian NMJ [57].

The addition of tubocurarine to the co-cultures led to an immediate halt in myotube contractions followed by a slow recovery of spontaneous activity, beginning with low frequency irregular spasms rather than the typical synchronised contractions observed in control conditions. Although, the re-establishment of contractile activity was observed (30 min) after the application of tubocurarine, the spontaneous myotube contractions did not return to a typical synchronous frequency until the co-cultures were restored to pre-intervention conditions. This observation is in line with reduced ability of ACh to open the ACh receptor in the presence of tubocurarine, acting as a competitive inhibitor of ACh [58], while αBTX functionally inactivates the AChRs. The ability of myotube contractile activity in the co-cultures to recover from tubocurarine is further evidence of functional neurotransmission in this *in vitro* NMJ system.

L-Glut (neuron-specific excitatory neurotransmitter) had an immediate stimulating impact on myotube contractility above that of control levels of spontaneous activity that returned to control levels within 10 min following MN stimulation with L-Glut. L-Glut catalyses the formation of γ-Aminobutyric acid (GABA), which enhances ACh release at the NMJ [59], thereby enhancing muscle contraction [60]. In summary, the responses to α-BTX, tubocurarine and L-Glut are all similar to that seen in *in vivo* NMJs indicating that in our system physiological NMJs were formed [60,61].

It is unclear whether the NMJs in our system are more human- or rat-like, which may limit clinical translation of data obtained with this cross-species culture system. Nevertheless, we have provided evidence that in our rat-human cross-species culture system functional NMJs are formed that are associated with a further differentiation of myotubes than we have seen previously with primary human muscle cells and embryonic stem cells [62], supporting the relevance of the system for use as a drug discovery platform to maintain neuromuscular interactions.

## 5. Conclusions

In summary, this study reports a platform that enables the analysis and modulation of NMJs, motor neuron and muscle simultaneously, which may provide a physiologically relevant and more effective translational model to enhance our understanding of NMJ physiology and pathophysiology. Physiological assessments of MU function were confirmed by spontaneous contraction profiles that were driven by motor neuron via the NMJ with inhibition and stimulation of the pre- and post-synaptic membrane. The similarity to *in vivo* contractility demonstrated by mature myotubes in this co-culture system improves research capabilities into neuromuscular physiology allowing for improved pathophysiological elucidation, diagnosis and treatment of diseases associated with neuromuscular dysfunction.

## Figures and Tables

**Table 1:** Complete growth media for skeletal muscle cell proliferation.

Growth Media Components/Company	Concentration	Catalogue #
Dulbecco’s Modified Eagle Media (DMEM) from Lonza	59% (*v*/*v*)	12–914F
Medium 199 with Earle’s Balanced Salt Solution from Lonza	20% (*v*/*v*)	12-119F
Heat-inactivated fetal bovine serum (FBS) from Thermo Fisher Scientific	20% (*v*/*v*)	10500-064
L-glutamine from Lonza	1% (*v*/*v*)	17-605E
Fetuin from fetal bovine serum from Sigma-Aldrich (St Louis, MO, USA)	25 μg/mL	F3004
Recombinant human basic fibroblast growth factor (FGFb) from Thermo Fisher Scientific	0.5 ng/mL	PHG0311
Recombinant human epidermal growth factor (EGF) from Thermo Fisher Scientific	5 ng/mL	PHG0311
Recombinant human hepatocyte growth factor (HGF) from Sino Biological Inc. (Beijing, China)	2.5 ng/mL	10463-HNAS
Recombinant human insulin from Sigma-Aldrich	5 μg/mL	91077C
Dexamethasone from Sigma-Aldrich	0.2 μg/mL	10103483
Gentamicin from Thermo Fisher Scientific	10 μg/mL	15710-049

**Table 2:** Primary antibodies.

Antibody/Company	Concentration	Catalogue #
Anti-Vesicular Acetylcholine Transporter (VAChT) from Merck Millipore (Burlington, MA, USA)	1:100	ABN100
Anti-Choline Acetyltransferase (ChAT) from Merck Millipore	1:100	AB144
Anti-Glial Fibrillary Acidic Protein (GFAP) from Sigma-Aldrich	1:100	G3893
Anti-Neurofilament Heavy (NFH) from Merck Millipore	1:100	AB5539
Anti-Synaptotagmin (Syt1) from Abcam (Cambridge, UK)	1:100	ab13259
Anti-Ryanodine Receptor 1 (RyR) from Merck Millipore	1:100	AB9078
Anti-Calcium channel L type DHPR alpha 2 subunit (DHPR) from Abcam	1:100	ab2864
Anti-Receptor-Associated Protein of the Synapse (Rapsyn) from Abcam	1:100	ab11423
Anti-Muscle-Specific Kinase (MuSK) from Abcam	1:100	ab92950
Anti-Beta Tubulin Class III Alexa Fluor^®^ 488 conjugate from Thermo Fisher Scientific	1:400	53-4510-82
α-Bungarotoxin, Alexa Fluor^®^ 647 conjugate from Thermo Fisher Scientific	1:400	B35450

## Supplementary Materials

The following are available online at https://www.mdpi.com/2077-0383/9/10/3238/s1, Video S1: Video S1 video recording of myotube contractions at Day 7.
