# Supplementary material for: A Novel Bioengineered Functional Motor Unit Platform to Study Neuromuscular Interaction
Source: J Clin Med. 2020 Oct 10;9(10):3238. doi: 10.3390/jcm9103238 (PMC7599749; doi:10.3390/jcm9103238)
Supplement: Supplementary file 1 [file jcm-09-03238-s001.zip › jcm-931668-supplementary.docx]

**Video S1 Video recording of myotube contractions at Day 7.**

Representative video of spontaneous innervated myotubes contracting within the co-culture system. Scale bar = 100 µm.
